# CRISPR/Cas9 editing of endogenous *banana streak virus* in the B genome of *Musa* spp. overcomes a major challenge in banana breeding

**DOI:** 10.1038/s42003-019-0288-7

**Published:** 2019-01-31

**Authors:** Jaindra N. Tripathi, Valentine O. Ntui, Mily Ron, Samwel K. Muiruri, Anne Britt, Leena Tripathi

**Affiliations:** 1 International Institute of Tropical Agriculture (IITA), Nairobi, Kenya; 2 Department of Plant Biology, University of California, Davis, CA USA

## Abstract

Presence of the integrated endogenous *banana streak virus* (eBSV) in the B genome of plantain (AAB) is a major challenge for breeding and dissemination of hybrids. As the eBSV activates into infectious viral particles under stress, the progenitor *Musa balbisiana* and its derivants, having at least one B genome, cannot be used as parents for crop improvement. Here, we report a strategy to inactivate the eBSV by editing the virus sequences. The regenerated genome-edited events of Gonja Manjaya showed mutations in the targeted sites with the potential to prevent proper transcription or/and translational into functional viral proteins. Seventy-five percent of the edited events remained asymptomatic in comparison to the non-edited control plants under water stress conditions, confirming inactivation of eBSV into infectious viral particles. This study paves the way for the improvement of B genome germplasm and its use in breeding programs to produce hybrids that can be globally disseminated.

## Abstract (web summary)

Jaindra Tirpathi et al. report a strategy for inactivating endogenous *banana streak virus* sequences in the plantain B genome using CRISPR/Cas9. They show that three-quarters of edited plants had no viral symptoms under stress conditions, providing an improved B genome germplasm for plantain and banana breeding.

## Introduction

B*anana streak virus* (BSV) is a plant pathogenic badnavirus of the family *Caulimoviridae*, affecting production of banana (*Musa* spp.). It is an unenveloped, non-covalently closed, bacilliform double-stranded DNA (dsDNA) virus with a monopartite genome of ~7.2–7.8 kb encoding three open-reading frames (ORFs). ORF1 encodes a small protein of unknown function that is associated with virions^1^. OFR2 encodes a protein of ~14 kDa, which is involved in virion assembly due to the presence of predicted N-terminal coiled-coil domain^1^. ORF3 contains domains associated with movement, the virus capsid, aspartic protease, reverse transcriptase, and ribonuclease H functions^2^.

BSV was first identified in West Africa in 1958 and is now reported in most banana and plantain growing countries^3^. Like other badnaviruses, BSV sequences integrate into the genome of *Musa* spp.^4^. BSV is a complex of different viruses belonging to the pararetroviruses and classified as endogenous pararetroviruses when they are integrated into a host genome. The BSV integrated in the banana host genome are known as endogenous BSV (eBSV).

Banana cultivars are polyploid clones derived from *Musa accuminata* (A genome) or/and *Musa balbisiana* (B genome). The eBSV sequences are mainly integrated in the B genome derived from *M. balbisiana*^5^. Many economically important sub-groups of banana, such as plantain (AAB), an important staple food in Africa, contain at least one B genome. Multiple copies of eBSV viral sequences have become integrated as direct and inverted tandem repeats at a single locus in the B genome of the host during viral infection^5^. When the banana plants are stressed, the eBSV recombines to produce a functional episomal viral genome and infectious viral particles and as a result the plant develops disease symptoms. In vitro culture for propagation and hybridization through conventional breeding may also trigger its activation. Consequently, BSV is considered as a major constraint in banana breeding programs, restricting the use of the diploid progenitor *M. balbisiana* or its derivants carrying a B genome as parents for introgression of desirable agronomic traits^6^. It also restricts germplasm movement of genotypes with the B genome worldwide due to this potential activation of eBSV into the episomal infectious form of virus. Therefore, it is crucial to design a strategy to irreversibly silence the latent eBSV in the B genome. In this study, CRISPR/Cas9-based genome-editing technology was applied to inactivate eBSV sequences in the host plantain genome. The genome-edited events of plantain Gonja Manjaya were generated with mutations in the targeted sites of integrated eBSV sequences in the host genome. Seventy-five percent (6 out of 8) of the edited events tested remained asymptomatic in comparison to the control non-edited plants under water stress conditions, confirming inhibition of eBSV and reversal of its ability to be converted into infectious viral particles in the edited lines.

## Results

### Confirmation of presence of eBSV in genome of Gonja Manjaya

**Fig. 1:**
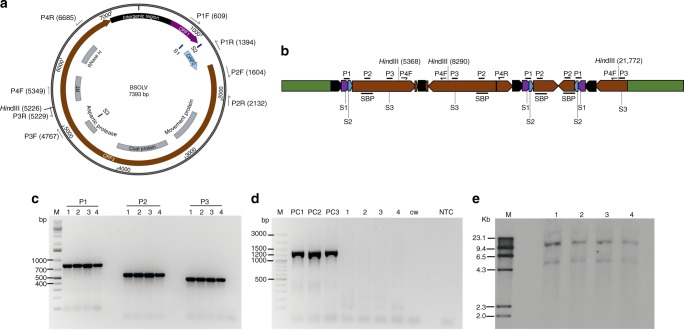
Schematic representation of *banana streak virus* (BSV) strain Obino l’Ewai (BSOLV) and molecular analysis of Gonja Manjaya plants. **a** Episomal BSOLV showing three open-reading frames (ORF) and gene annotation in ORF3 (GenBank accession KJ013506). The colors purple, light blue, brown, and black indicate ORF1, ORF2, ORF3, and the intragenic region of BSOLV, respectively; P1–P4 show the position of primer pairs; S1–S3 indicate target sites for gRNA1, gRNA2, and gRNA3, respectively. **b** Endogenous BSV strain Obino l’Ewai (eBSOLV) integrated in banana genome (adapted from ref. ^5^). The duplication of BSV sequences, either complete or partial in the same or opposite direction, within the integrated eBSOLV are shown in the diagram. Green indicates banana genome; SBP indicates position of probe used for Southern hybridization; P1–P4 represent PCR primer pair locations. **c** PCR amplification to confirm the presence of BSOLV and/or eBSOLV in Gonja Manjaya plantlets. 1‒4, in vitro mother plantlets of Gonja Manjaya; PCR products using P1–P3 primers are labeled on the top of the gel picture; M molecular marker. **d** PCR analysis to confirm the absence of episomal BSOLV in in vitro mother plantlets of Gonja Manjaya used for genome editing. PC1 and PC2, symptomatic field plants of plantain Agbagba; PC3, symptomatic Gonja Manjaya plant from glasshouse under stress condition; CW, asymptomatic in vitro plantlet of Cavendish Williams as negative control; NTC, no template control. **e** Southern blot hybridization to confirm the presence of eBSOLV in Gonja Manjaya plantlets. Genomic DNA for Southern blot was digested with *Hin*dIII. The same four individual Gonja Manjaya plants (1‒4) were used for PCR analyses and Southern hybridization

Gonja Manjaya, a false horn plantain (AAB genome) commonly grown in East and Central Africa, was initiated from an asymptomatic field plant and used in this study to knockout the integrated sequences of eBSV. The BSV strain Obino l’Ewai (BSOLV), is widely distributed among plantain plantations in Africa. BSOLV exhibits in two forms, episomal (Fig. 1a, GenBank accession KJ013506) and eBSOLV^5^ (Fig. 1b, GenBank accession AP009334) integrated in a recombined form at a single locus in the B genome of plantain. The presence of BSOLV or eBSOLV sequences was confirmed in the mother stock of in vitro plantlets by PCR analysis using the three different primer pairs (P1–P3) (Fig. 1c), which amplify both the endogenous and episomal forms of the virus. The presence of the episomal form of BSOLV was ruled out by PCR analysis using primer pair P4 that specifically amplifies BSOLV but not the integrated eBSOLV^7^. The P4 primer pair (Fig. 1a) binds in the ORF3 region of BSOLV and generates a 1336 bp fragment in the presence of the replicating viral genome. This primer pair also binds to eBSOLV, but the position and orientation of the two primers’ binding precludes PCR amplification (Fig. 1b). The amplified fragment diagnostic for the episomal BSOLV was also observed in the symptomatic plants of plantain Agbagba, collected from the field as positive controls (PC1–2) and a symptomatic glasshouse plant of Gonja Manjaya (PC3). However, no amplification was observed in any of the in vitro Gonja Manjaya plantlets, similar to the asymptomatic control plant of Cavendish Williams (AAA) (Fig. 1d).

The presence of eBSOLV in the host genome was further confirmed by Southern blot analysis using a BSOLV-specific probe (termed SBP, Fig. 1b–e). The genomic DNA was restricted with *Hin*dIII, which has only one site in the episomal BSOLV and three sites in the eBSOLV (Fig. 1a, b). As expected based on the genomic sequence of eBSOLV integrated in the reference genome of *M. balbisiana* (Accession AP009334) (Fig. 1b), two hybridization signals (about 13.5 and 5.4 kb) were observed in all four samples tested (Fig. 1e). As predicted, a band of 13.5 kb was obtained by restriction of two *Hin*dIII sites within eBSOLV DNA sequence and a second fragment of about 5.4 kb was obtained by cleavage at one restriction site in eBSOLV DNA sequence and a second site in the flanking genome of Gonja Manjaya bordering the integrated site.

gThe in vitro plantlet 3 with the expected two bands on Southern hybridization (Fig. 1e) and PCR product characteristics of integrated eBSOLV, but no episomal BSOLV (Fig. 1d) was used for developing embryogenic cell suspensions. These cell suspensions were used to generate genome-edited plants after confirming the absence of episomal BSOLV and the presence of integrated eBSOLV in their genome by PCR analysis.

### Preparation of CRISPR/Cas9 plasmid construct

**Fig. 2:**
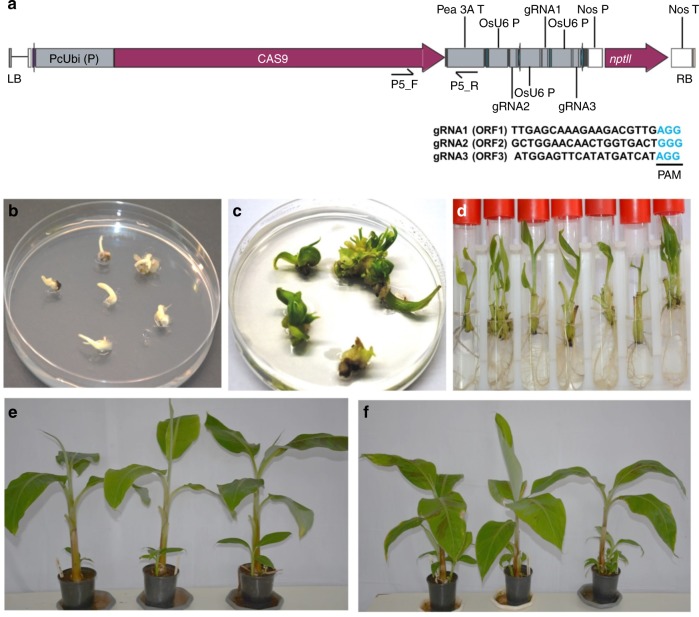
Regeneration of genome-edited events by delivering CRISPR/Cas9 construct through *Agrobacterium*-mediated transformation of cell suspension of Gonja Manjaya. **a** Schematic representation of T-DNA region of binary vector pMR215-BSV1 showing the positions of three gRNAs. LB, left border; RB, right border; PAM, protospacer adjacent motif. Sequences of the gRNAs are also indicated. **b** and **c** Germination of *Agro*-infected embryos in selective medium containing kanamycin. **d** Well-rooted plantlets in proliferation medium. **e** Genome-edited Gonja Manjaya plants in glasshouse. **f** Non-edited wild type control Gonja Manjaya plants in glasshouse

The gRNAs were designed for each CRISPR target from the most conserved sequences of the BSOLV (GenBank accession KJ013506) and eBSOLV (GenBank accession AP009334 and HE983609). The gRNAs [gRNA1 (ORF1)-TTGAGCAAAGAAGACGTTG, gRNA2 (ORF2)-GCTGGAACAACTGGTGACT and gRNA3 (ORF3)-ATGGAGTTCATATGATCAT] were designed for the three CRISPR targets (S1–S3) in the three ORFs of BSOLV and eBSOLV (Fig. 1a, b). All the three gRNAs were integrated into the T-DNA region of a Cas9 expression vector pDe-Cas9-Kan^8^ to generate the plasmid, pMR215-BSV1 (Fig. 2a).

### Generation and validation of transgenic events

The CRISPR/Cas9 construct was delivered into the embryogenic cell suspensions of Gonja Manjaya via *Agrobacterium tumefaciens*. A total of 100 kanamycin-resistant, well-rooted plantlets were generated (Fig. 2b–d). Fifty random regenerants were selected and screened for the presence of the *Cas9* gene by PCR using primers P5 (Fig. 2a, Supplementary Table 1). The transgenic events displayed a 906 bp fragment, as expected, confirming the presence of the *Cas9* gene (Supplementary Figure 1a).

The well-rooted plantlets of *Cas9* PCR-positive events and non-transgenic control were acclimatized and potted in the greenhouse (Fig. 2e–f).

### Molecular analysis to confirm editing in ORF1 and ORF2

Three gRNAs (targeting sequences S1 in ORF1, S2 in ORF2, and S3 in ORF3) were introduced into triploid Gonja Manjaya (AAB). The eBSOLV is known to integrate at a single locus on chromosome 1 of the B genome based on the *M. balbisiana* sequence (GenBank accession AP009334 and HE983609). There is only one infectious allele of eBSOLV^6^. BSOLV sequences are duplicated in the same or opposite direction resulting in several copies of ORFs (either complete or partial) within the integrated eBSOLV (Fig. 1b). As a result of these duplications, each guide (S1–S3) has three targets in the BSOLV sequences integrated in the plant genome (Fig. 1b).

Twenty independent transgenic events (1, 4, 7, 17, 29, 30, 33, 36, 48, 53, 66, 72, 76, 78, 81, 85, 87, 94, 97, and 100), confirmed for the presence of the *Cas9* gene, were analyzed for a shift in the size of PCR amplicons as an indication for mutations in target sites S1 and S2. PCR analysis was performed using primer pair P1 flanking the gRNA1 (in S1 targets) and gRNA2 (in S2 targets) (Fig. 1, Supplementary Figure 1b). The amplicons of several of these independently derived events showed a predicted band shift of 197 bp, suggesting deletions in the region between S1 and S2 as expected when Cas9 cuts both target sites simultaneously (Supplementary Figure 1b). Seven (36, 66, 81, 85, 87, 94, and 97) out of 20 events showed shorter amplicons (band shift by 197 bp) in addition to amplicons similar in size to those of wild type control plants suggesting that they might be heterozygous for mutations in all the copies of target sites S1 and S2. Two events (81 and 85) showed a third amplicon with a smaller deletion indicating these plants contained additional genome rearrangements. Two other events (48 and 76) had only the smaller amplicons suggesting all target sites might have the same mutation, while the remaining nine events (1, 4, 7, 17, 29, 53, 72, 78, and 100) showed amplicons similar to the wild type control plant (Supplementary Figure 1b), indicating that they might have no indels or small indels.

A T7 endonuclease I (T7E1) assay was performed to check for the presence of small indels in the nine transgenic events (1, 4, 7, 17, 29, 53, 72, 78, and 100), which did not show any shift in the size of amplicon compared with the wild type control. T7E1-digested fragments were detected in all the events except for events 7 and 78, confirming the mutated sites in these events (Supplementary Figure 1c).

**Fig. 3:**
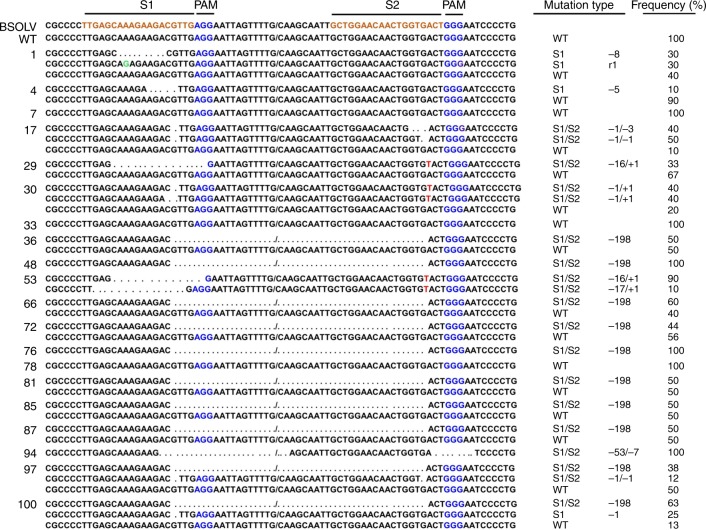
Sequence analysis of genome-edited plants of Gonja Manjaya to detect mutations in S1 and S2 target sites. Nucleotides in brown represent the target sites and blue nucleotides indicate protospacer adjacent motif (PAM). Dotted lines (…) represent deletions, nucleotides in red indicate insertion, and green nucleotides indicate replacement. BSOLV, sequence of BSV strain Obino l’Ewai; WT, sequences same as wild type non-edited control Gonja Manjaya; 1–100, 20 independent transgenic events. The mutations in S1, S2, and S1/S2, number of indels as deletions (−), insertions (+) or replacements (r) and percentage of clones of transgenic event showing same mutations are indicated on the right side panel. The PCR products amplified using primer P1 (see Fig. 1b) were purified, cloned, and 10 individual clones per event were analyzed for mutations at target sites S1 and S2 by Sanger sequencing

All the 20 independent events (1, 4, 7, 17, 29, 30, 33, 36, 48, 53, 66, 72, 76, 78, 81, 85, 87, 94, 97, and 100) tested by PCR analysis were further analyzed for targeted mutations by sequencing of target sites S1 and S2. The PCR products amplified using P1 primers were purified and cloned, and 10 individual clones per event sequenced. Sequencing of the cloned PCR products further confirmed the targeted mutations in all the edited events except for 3 events (7, 33, and 78) confirming a mutation efficiency of 85% in the targeted site S1 and S2 of transgenic plants (Fig. 3). All 17 of these independently derived mutant plants showed either only deletions (∆1 bp to ∆198 bp) or deletions (∆1 bp to ∆17 bp) accompanied by insertion (+1) at the predicted Cas9 cleavage site, 3 bp upstream of PAM motif (Fig. 3). Ten (36, 48, 66, 72, 76, 81, 85, 87, 97, and 100) out of 20 events tested showed deletions of 198 bp, suggesting that Cas9 cuts 4 bp upstream of the PAM for gRNA1 and 3 bp upstream of the PAM for gRNA2. Simultaneous cutting at S1 and S2 site was apparently repaired to generate the deletion of 198 nucleotides in a coding region, eliminating 66 codons. In other events (1, 4, 17, 29, 30, and 53), the deletions were smaller. Events 17 and 94 showed small deletions at both target sites, indicating Cas9 cleavage and independent mutagenic repair events at two nearby target sites in the same eBSOLV. Events 29, 30, and 53 also showed single nucleotide insertion in addition to deletions at one target site. Only one nucleotide replacement was observed in event 1 (Fig. 3). The majority of edited events (15/17) showed mutations at both S1 and S2 targeted sites. All the edited plants contained indels resulting in mutations with the ability to prevent proper transcription of eBSOLV genes or/and translational to non-functional viral proteins.

Only two edited events (48 and 76) showed the same indels in all the 10 clones sequenced, which was in agreement with the result of band shift PCR analysis showing one amplicon (Fig. 3). The other events showed 2‒3 different versions of sequences of the target sites indicating that these events might be chimeric, with mutations happening at different stages of plant regeneration, or indicating differences in mutations at the various copies of the target sites in a single eBSOLV. The sequencing result of events 48 and 76 also confirmed knockout of all the three copies of target sequences of eBSOLV from the plantain genome.

Event 76 was further tested for uniformity and stability of mutations. Sequencing of three different leaves from the same plantlet showed the same deletion (∆198 bp) indicating uniform mutations in event 76. The sequencing of daughter plants of event 76 after many sub-culturing cycles also showed the same deletions (∆198 bp), thus, confirming the stability of mutations during multiple rounds of vegetative propagation.

All four different wild type non-transgenic control plants, generated from the same embryogenic cell line used in the experiments along with the mutant plants, showed wild type sequences with no indels confirming that all the mutations among the different transgenic events were due to the action of CRISPR/Cas9.

### Molecular analysis to confirm edits in ORF3 of eBSOLV

**Fig. 4:**
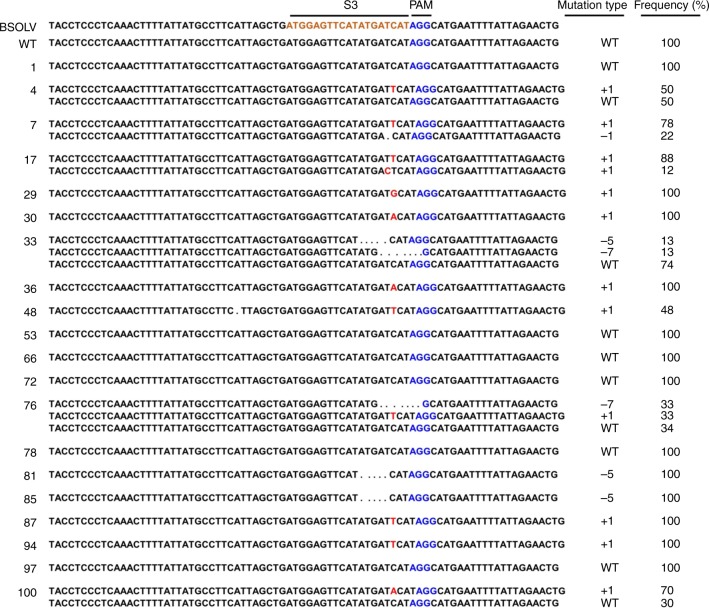
Sequence analysis of genome-edited plants of Gonja Manjaya to detect targeted mutations in S3 target site. Nucleotides in brown represent the target site and blue nucleotides indicate PAM. Dotted lines (…) represent deletions, nucleotides in red indicate insertion. BSOLV, sequence of BSV strain Obino l’Ewai; WT, wild type non-edited control Gonja Manjaya; 1‒100, independent transgenic events. The number of indels as deletions (−) or insertions (+) and percentage of clones of transgenic event showing same mutations are indicated on the right side of the figure. The PCR products amplified using primer set P3 (see Fig. 1b) were purified, cloned, and 10 individual clones per event were analyzed for mutations at target site S3 by Sanger sequencing

The PCR product for all 20 events (1, 4, 7, 17, 29, 30, 33, 36, 48, 53, 66, 72, 76, 78, 81, 85, 87, 94, 97, and 100) in which the target S3 site was amplified using the P3 primer pair were purified and cloned. Ten individual clones per event were analyzed for mutations at the S3 target site by DNA sequencing (Fig. 4). The sequence analysis showed a variety of mutations (deletion or insertion or deletion accompanied by insertion) in 14 events (4, 7, 17, 29, 30, 33, 36, 48, 76, 81, 85, 87, 94, and 100) while the remaining six events (1, 53, 66, 72, 78, and 97) did not show any indels in the S3 site (Fig. 4) indicating editing efficiency of 70% at site S3. For this target site, only smaller deletions ranging between 1 and 7 bp and a single bp insertion were observed. Most of the mutations were observed at 3 bp upstream of PAM, as expected. All the 14 edited events showed indels leading to reading frame shift and/or other mutations that have potential to inactivate the eBSOLV genes (Fig. 4).

Out of the 20 events analyzed for editing in three different target sequences, 11 events (17, 29, 30, 36, 48, 76, 81, 85, 87, 94, and 100) showed editing in all the targeted sites (S1–S3), two events (7 and 33) showed editing only in S3 site with no indels in S1 and S2 site, and four events (53, 66, 72, and 97) showed mutations in S1/S2 sites but no indels in S3 site. Event 1 showed indels at only the S1 site and no indels in S2 and S3 sites, whereas event 4 showed mutations in S1 and S3 sites but no indels in S2 site. There was only one event (78) that did not show indels in any of the targeted sites indicating a very high mutation efficiency of 95% using three gRNAs.

### Off-target analysis

In searches for potential off-target mutations for each gRNA using the CRISPR web-tool Breaking-Cas (http://bioinfogp.cnb.csic.es/tools/breakingcas), a total of 66 potential off-targets were discovered. Because plantain contains two genomes, A and B, the gRNA + PAM sequences were blasted against the respective banana genomes (http://banana-genome-hub.southgreen.fr). Putative 58 off-target sites were identified in the A-genome from *M. accuminata* and 40 in the B-genome from *M. balbisiana* (Table 1). Based on the banana genome, all these potential off-target sites had matches of 63‒95% to the gRNA+PAM sequences (Supplementary Table 2). Seven putative off-target loci with high sequence similarity to the gRNAs or/and containing a Cas9 PAM sequence (NGG), were selected for sequencing and detailed examination of potential off-target effects in nine edited events (1, 17, 48, 66, 76, 81, 85, 87, and 97) (Table 2). Of the seven selected genomic loci, only one of them contained PAM motif (NGG), which could potentially be targeted by gRNA–Cas9.

**Table 1:** Number of potential off-target sites for mutations in A and B genomes of plantain for the three CRISPR targets in eBSOLV integrated in Gonja Manjaya (AAB)

gRNA sequence from eBSOLV + *PAM*	Number of potential off-targets	
	A genome	B genome
TTGAGCAAAGAAGACGTTG*AGG*	11	9
GCTGGAACAACTGGTGACT*GGG*	13	6
ATGGAGTTCATATGATCAT*AGG*	34	25

The PAM sequences are shown in italics

**Table 2:** Selected potential off-target sites used for sequence analyzing of genome-edited events of Gonja Manjaya (AAB)

gRNA sequence from eBSOLV + *PAM*	A genome			B genome		
	Potential off-target site (Gene ID)	Identity (%)	Sequence alignment	Potential off-target site (Gene ID)	Identity (%)	Sequence alignment
TTGAGCAAAGAAGACGTTG*AGG*	Ma05_t06600.1	77.27	GAGCAAAGAAGACGTTGA | | | | | | | | | | | | | | | | | GAGCAAAGAAGAAGTTGA	ITC1587_Bchr5_T12273	77.27	GAGCAAAGAAGACGTTGA | | | | | | | | | | | | | | | | |GAGCAAAGAAGAAGTTGA
GCTGGAACAACTGGTGACT*GGG*	Ma05_t31620.1	95.65	TGCTGGAACAACTGGTGACT*GGG* | | | | | | | | | | | | | | | | | *| | |* TGCTGAAAAATCTGGTGACT*GGG*	ITC1587_Bchr4_T08704	63.64	GCTGGAACAACTGGT | | | | | | | | | | | | | | | GCTGGAACAACTGGT
	Ma01_t10610.1	86.36	CTGGAACAACTGGTGACTGG | | | | | | | | | | | | | | | | | | CTGGTCCAACTGGTGACTGG			
ATGGAGTTCATATGATCAT*AGG*	Ma08_t27880.1	86.36	ATGGAGTTCATATGATCATA | | | | | | | | | | | | | | | | | | ATGGAGACCATATGATCATA	ITC1587_Bchr1_T00896	86.36	TGGAGTTCATATGATCATAG | | | | | | | | | | | | | | | | | | TGGAGTTCATATGTTTATAG

Seven putative off-target loci containing high sequence similarity to the gRNAs or/and PAM sequence (NGG) were selected for sequencing of nine genome-edited events. The PAM sequences are shown in italics

Sequencing of seven potential off-target sites in nine events showed point mutation at one putative off-target locus (Ma01_t10610.1) in the genome of just one event (72). This off-target site was in the A genome sharing 86% nucleotide identity with the target site. The site did not contain the same PAM sequence (GGG) as the intended target, but the guide might potentially be able to recognize an NAG PAM sequence in close proximity. These results indicate a very low probability for potential off-target mutations.

### Glasshouse evaluation of genome-edited plants

Eight genome-edited events (17, 48, 66, 76, 81, 85, 87, and 97) along with three wild type control plants of Gonja Manjaya were potted, acclimatized, and grown in the greenhouse. The genome-edited plants and wild type control plants, developed from the same embryogenic cell line, were found to be phenotypically similar with no growth abnormalities (Fig. 2e, f).

**Fig. 5:**
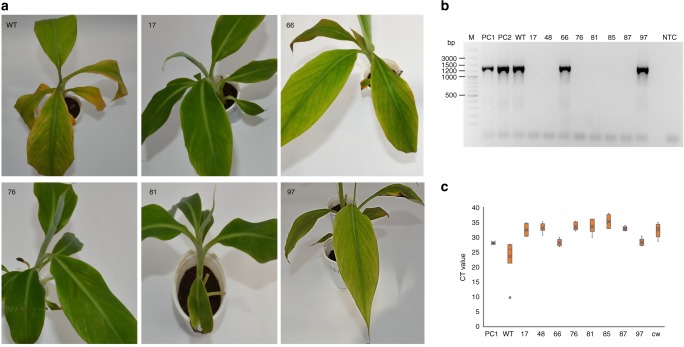
Evaluation of genome-edited and wild type non-edited control plants of Gonja Manjaya for induction of BSV symptoms under water stress conditions. Two-month-old plants were subjected to water stress for 2 weeks. Disease symptoms as chlorosis or yellow streaks were recorded at the end of the stress period and pictures were taken. **a** Pictures of asymptomatic genome-edited plants (17, 76, and 81), symptomatic-edited plants (66 and 97), and wild type control plants (WT). **b** PCR diagnostic to detect activation of episomal BSOLV in genome edited and control plants under water stress conditions. **c** qPCR analysis to detect episomal BSOLV in genome edited and control plants under water stress conditions. PC1‒2, symptomatic plant of Agbagba from field as positive control; WT, wild type non-edited control Gonja Manjaya plant under stress conditions; 17, 66, 76, 81, 97, edited plants under stress conditions; CW, asymptomatic in vitro plantlet of Cavendish Williams as negative control; NTC no template control. For PCR and qPCR, leaf samples from three symptomatic wild type non-edited control plants (WT) of Gonja Manjaya were pooled for DNA extraction. Similarly, the leaves from three replicates for symptomatic and asymptomatic-edited events were pooled for PCR and qPCR. CT values were presented as means and standard error of six technical replicates from two independent experiments

After 2 months, the plants were subjected to water stress for 2 weeks and observed daily for development of BSV symptoms for 60 days. Disease symptoms such as broken or continuous streaks of yellow, chlorotic, black, or brown color on the leaf appeared in all three of the wild type control plants tested, as would be expected if there was activation of eBSOLV into episomal infectious BSOLV (Fig. 5a). However, six (17, 48, 76, 81, 85, and 87) out of eight genome-edited events tested remained asymptomic and the remaining two events (66 and 97) showed only moderate symptoms (Fig. 5). The presence of the infectious episomal form of BSOLV was confirmed by PCR using primers^7^ (employed in Fig. 1d) that detect BSOLV but not eBSOLV. PCR analysis showed amplification of BSOLV in symptomatic plants of genome-edited plants 66 and 97 and in the stressed wild type Gonja Manjaya plant, as well as the symptomatic field plant sample of plantain Agbagba (Fig. 5b). However, BSOLV was not detected in asymptomatic genome-edited (17, 48, 76, 81, 85, and 87) events (Fig. 5b). This result was further validated by qPCR using the same primers^7^. In these analyses, the cycle threshold (CT) values for all the samples tested ranged from 23 to 35, whereas the stressed wild type Gonja Manjaya plant showed a lower CT of 23.6 (Fig. 5c). The symptomatic field plant of Agbagba (PC1) and symptomatic-edited plants (66 and 97) showed a CT of about 28 confirming the presence of BSOLV. The asymptomatic-edited plants (17, 48, 76, 81, 85, and 87) showed CT values similar or higher to the asymptomatic plants of Cavendish Williams (CW) confirming the absence of BSOLV (Fig. 5c).

The events 66 and 97 showed mutations in S1 and S2 target sites but no mutations in the S3 target site, however the asymptomatic edited events (17, 48, 76, 81, 85, and 87) showed indels in all the targeted sites suggesting either that knockouts in all the ORFs are important for complete inactivation of the virus, or that mutations in S3 are especially effective. This result indicates that targeted mutagenesis of eBSOLV can lead to its stable inactivation, preventing the emergence of infectious BSV.

## Discussion

Banana and plantains are important staple food crops cultivated across tropical and subtropical countries. Genetic improvement of banana by conventional breeding is key for the development of improved varieties with disease and pest resistance and higher yields. BSV is one of the major challenges in banana breeding and dissemination of plantain hybrids due to the presence of integrated eBSV in the B genome^4^. These proviruses can be activated into the infectious episomal BSV by various stress conditions. Thus, major epidemics caused by BSV are not due to natural transmission through mealy bugs or through use of infected planting materials, but, rather due to activation of eBSV^9^ under stress conditions, such as in vitro propagation, hybridization or/and unfavorable conditions like water and temperature stress. As a result, BSV has become a hurdle to overcome in plantain breeding. Breeders cannot use diploid *M. balbisiana* (BB) in breeding programs, even though it has good attributes like hardiness, strong root system, and tolerance to biotic and abiotic stresses. Therefore, it is necessary to make the B genome of banana free of the activatable sequences of eBSV. With this in mind, in this study we knocked out the integrated eBSV by targeted mutagenesis using CRISPR/Cas9.

CRISPR-mediated genome editing for plant virus resistance is mainly reported for ssDNA viruses like geminiviruses^10–12^ and ssRNA viruses^13,14^. CRISPR/Cas9 technology can also be applied to either dsDNA viruses or retroviruses that have dsDNA as part of their life cycle^15^. Currently, this has not been demonstrated for any plant viruses, however, application of CRISPR/Cas9 to interrupt both episomal and integrated dsDNA viruses has been demonstrated in many human viruses^16–18^. HIV is a retrovirus that integrates its viral DNA into the host DNA and has potential to reactivate causing the disease, HIV-AIDS. Recently, inactivation of HIV-1, was achieved by knocking out proviral DNA integrated in latently infected cells by targeting long terminal repeat flanking sequences or multiple regulatory genes within the HIV-1 provirus^19,20^. Similarly, in this study, we used CRISPR/Cas9 to knockout the integrated dsDNA of BSV from the banana genome using gRNAs targeting multiple repeats of all the three ORFs of BSV (Fig. 1).

Our results showed high mutation efficiency of 95% in the targeted sites (S1–S3) using multiple gRNAs, which is in agreement with a recent study in which 100% editing frequency was reported for banana using a polycistronic gRNA plasmid construct targeting the *PDS* gene^21^. In our study, the frequency of mutations differed between the target sites. About 85% of events (17/20) showed indels in the S1 and S2 target sites and 59% of these mutants showed big deletions of 198 bp as a result of simultaneous cleavage of both target sites by Cas9. Simultaneous action of two gRNAs targeting sequences in close proximity has been shown to generate large deletions in other plant species as well^22–25^. A comparatively lower editing efficiency of 70% with small indels was observed with a single gRNA-targeting site S3. Similarly, a lower editing efficiency of 59% with small indels was also reported previously in banana using a single gRNA targeting the *PDS* gene^26^.

The sequencing of target sites S1 and S2 of edited events 48 and 76 showed the same indels in all the clones sequenced, confirming knockout of all the three copies of ORF1 and ORF2 in the integrated eBSOLV sequence from the plantain genome. The eBSOLV has multiple copies of ORFs inserted at the same locus on chromosome 1^5^ (Fig. 1). All the edited events showed indels with mutations in the targeted sites in ORFs resulting in the potential defects in gene transcription or mRNA translation.

Our results confirmed the uniform and stable inheritance of targeted mutations by CRISPR/Cas9 to clonal progeny. A similar result of stable inheritance of a disease resistance trait was observed in the field-testing of transgenic banana^27^.

A common limitation of Cas9-mediated genome editing is the possibility of off-target mutation of regions similar to the target site^28^. In order to minimize the off-target effects in this study, the gRNAs were selected based on their specificity to the target site and minimal potential off targets in the *Musa* genome. Our results showed very low potential off-target site mutations indicating that CRISPR/Cas9-induced mutagenesis in banana is precise and efficient.

The evaluation of eight edited events under water stress conditions in the glasshouse showed BSV symptoms in only two events, whereas the other six events remained asymptomatic. PCR and qPCR analysis of these plants confirmed the activation of eBSOLV into infectious episomal BSOLV in the two symptomatic-edited events and stressed control wild type plants, whereas no infectious virus was detected in the six asymptomatic-edited events. The qPCR assay is reported to be specific for detection of episomal BSOLV^7^.

The asymptomatic events have indels in all the three ORFs, whereas symptomatic events had deletions only in ORF1 and ORF2. This result suggests that ORF3, encoding essential proteins, such as movement protein, coat protein, aspartic protease, reverse transcriptase, and ribonuclease H, may play a crucial role in symptom development. ORF3 encodes a polyprotein, which is cleaved post-translationally by aspartic protease^29^ into functional versions of each of the ORF3-encoded proteins. In this study, the mutations created in the aspartic protease gene should have disrupted the functions of the protease and thus, the functions of other ORF3 genes as well. However, there is need for multiple knockouts in all three ORFs to completely prevent activation of eBSV as indels only in one ORF may result in partial inactivation and might allow viral escape. Although the edited plants with only mutations in the ORF3 region were not tested for activation of eBSOLV in this study, plants with mutations only in ORF3 may also exhibit partial inactivation of the virus as ORF2 encodes for a protein that is involved in virion assembly^1^. Therefore, it appears that simultaneous generation of mutations in all three ORFs is an important strategy for completely inactivating the provirus and preventing the occurrence of symptoms. Indeed, regarding HIV-1, a retrovirus whose provirus is similar to eBSV, studies have demonstrated the strong suppression of HIV-1 using a multiplex approach^19,20,30,31^.

BSV is a complicated virus and mechanisms for its integration, activation, and subsequent episomal infection are still largely unknown^5^. There is only one infectious allele^6^ of eBSOLV integrated in the chromosome 1 of B genome of Gonja Manjaya. However, three or more copies (complete or partial) of each ORF were present either in the same or opposite orientation within the eBSOLV sequences (Fig. 1b).

It is interesting that a few events showing indels in some clones and wild type sequence in other clones showed a knockout phenotype with no BSV symptom development under stress conditions. Although this triploid genome carries only a single copy of the integrated sequence, that integrated sequence carries three copies of each target, in various orientations. The symptom-free plants may carry mutations copies of the target that are essential for production of a functional episomal virus. Further analysis of these plants may enable us to distinguish between essential and disposable copies of the target site.

In summary, we have successfully applied the CRISPR/Cas9 system to edit the integrated sequence of eBSOLV in plantain cultivar Gonja Manjaya. Our results also demonstrated that multiplexing of CRISPR/Cas9 technology is very efficient in creating precise deletions in banana. Although preliminary results showed stable mutations and silencing of the reactivation of eBSOLV, more robust evaluation of promising edited plants in the field for several generations and with new BSV infections, due to natural transmission through mealy bugs in the field conditions, is required.

This is the first report to our knowledge to demonstrate the knockout of integrated endogenous DNA sequences of the pararetrovirus in a host plant genome. We have demonstrated CRISPR/Cas9-based targeted mutagenesis can permanently inactivate endogenous eBSV, and presents a promising model for the inactivation of other endogenous viral genomes. This study paves the way for editing of banana germplasm with B genome(s) that can be used as one or both parents in the breeding programs. This is a particularly important strategy for both improving plantains and enabling global dissemination of the resulting hybrids with improved B genome.

## Methods

### Plant material

Plantain cultivar Gonja Manjaya (genome AAB), showing no symptoms of BSV under field conditions, was collected from the field in Uganda and used in this study. The in vitro culture was established from the sucker collected from banana field as described^32^. The outer layer of leaves and corm tissue were removed from the suckers to obtain an explant of 5 × 10 cm long. The explants were washed with water and surface sterilized by immersing in 70% ethanol for 5 min followed by soaking in 15% sodium hypochlorite solution with few drops of Tween 20 for 10 min. After surface sterilization, the explants were rinsed 3–4 times with sterile distilled water. The explants were further trimmed by removing the outer sheaths to obtain the size of 1 × 1 cm. The explant was divided into two pieces through the meristem and cultured in the proliferation medium [4.4 g L^−1^ Murashige and Skoog medium including vitamins (Duchefa, M0222), 10 mg L^−1^ ascorbic acid, 5 mg L^−1^ 6-benzylaminopurine, 30 g L^−1^ sucrose, 3 g L^−1^ gelrite, pH 5.8] at 26 ± 2 °C under 16 h photoperiod furnished with fluorescent tube providing light of 94 μmol m^−2^ s^−1^. The explants were transferred to fresh medium every 2 weeks. Once the in vitro culture was initiated, it was maintained by sub-culturing the shoots to fresh medium every 4 weeks. The shoots regenerated on proliferation medium were transferred to rooting medium [4.4 g L^−1^ Murashige and Skoog medium including vitamins (Duchefa, M0222), 10 mg L^−1^ ascorbic acid, 1 mg L^−1^ indole-3-butyric acid, 30 g L^−1^ sucrose, 3 g L^−1^ gelrite, pH 5.8] for development of well-rooted plantlets.

The in vitro mother stock plantlets were checked for the presence of BSOLV or eBSOLV in the host by PCR using three pairs of primers P1–P3 (Supplementary Table 1, Fig. 1). Genomic DNA was extracted from leaves of four in vitro plantlets of Gonja Manjaya plants using CTAB^33^. About 500 mg of fresh leaf samples were ground to fine powder using mortar and pestle, and resuspended in 500 μL of extraction buffer (100 mM Tris–HCl, pH 8.0; 20 mM EDTA pH 8.0; 1.4 M NaCl; 2% PVP-10; 0.8% cetyltrimethylammonium bromide, and 0.001% mercaptoethanol). The samples were vortex vigorously, incubated at 65 °C for 30 min and then centrifuged at 15,000 rpm for 10 min. The supernatant was collected and mixed with an equal volume of chloroform:isoamyl alcohol (24:1) and centrifuged at 15,000 rpm for 10 min. This step was repeated. After a second chloroform:isoamyl alcohol extraction, the supernatant was collected and mixed with an equal volume of pre-chilled isopropanol and incubated at room temperature for 10 min for precipitation of DNA. Nucleic acids were pelleted by centrifugation at 15,000 rpm for 10 min. The DNA pellets were washed with 70% ethanol, air-dried and resuspended in 100 μL of TE buffer containing RNase. DNA was reprecipitated with 1 mL of absolute ethanol for 30 min and centrifuged at 12,000 rpm for 10 min. The supernatant was discarded and DNA pellet was air-dried and dissolve in 100 μL nuclease-free water. The concentration and quality of the DNA was checked with nanodrop.

PCR was performed in a 20 µL reaction volume containing 1 µL genomic DNA (20 ng µL^−1^), 10 µL of HotStarTaq master mix, 1 µL of 10 µM of each primer, and 7 µL nuclease-free water. PCR reaction conditions were: initial denaturation step at 95 °C for 15 min, followed by 34 cycles of denaturation at 94  °C for 30 s, annealing at 57 °C for 30 s, extension at 72 °C for 1 min, and final extension at 72 °C for 10 min. PCR amplicons were resolved on 1% agarose gel.

The presence of episomal BSV was ruled out by PCR amplification using primer set P4 (Supplementary Table 1), which amplifies only episomal BSOLV and not integrated eBSOLV^7^ (Fig. 1). The symptomatic field grown plants of plantain Agbagba (AAB) showing BSV symptoms and symptomatic Gonja Manjaya plants from the glasshouse were used as positive controls and asymptomatic in vitro plantlet of Cavendish Williams (AAA) was used as a negative control. PCR was performed with 20 ng of genomic DNA with an initial denaturation step at 95 °C for 15 min, followed by 34 cycles of denaturation at 94 °C for 30 s, annealing at 55 °C for 30 s, extension at 72 °C for 1 min, and final extension at 72 °C for 10 min. After amplification, 5 µL of PCR product was resolved on 1% agarose gel stained with gel red.

The integration of eBSOLV was further confirmed by Southern blot hybridization. The genomic DNA of four in vitro plantlets of Gonja Manjaya was restricted with *Hin*dIII, resolved on 0.8% agarose gel, and transferred to a nylon membrane. *Hin*dIII cuts at a single site within the BSOLV genome and three sites in eBSOLV (Fig. 1a, b). The blot was hybridized with DIG-labeled probe specific to the ORF3 region of BSOLV (Fig. 1) and detected by CDP star chemiluminiscent substrate.

After validation for the presence of eBSOLV and absence of BSOLV, the in vitro plantlet 3 was multiplied and used for developing embryogenic cell suspensions using multiple buds following the method as described^32^. The embryogenic callus was induced by culturing slices (1–2 mm thick) of multiple buds on callus induction medium [4.4 g L^−1^ Murashige and Skoog medium including vitamins (Duchefa, M0222), 10 mg L^−1^ ascorbic acid, 1 mg L^−1^ 2,4-dichlorophenoxyacetic acid, 0.2 mg L^−1^ zeatin, 30 g L^−1^ sucrose, 3 g L^−1^ gelrite, pH 5.8) in the dark at 26 ± 2 °C for 3–6 months. The embryogenic calli were transferred to liquid callus induction medium in conical flasks and incubated in the dark at 26 ± 2 °C, with agitation at 90 rpm. Once the homogenous embryogenic cell suspensions were established, they were maintained and multiplied by regular sub-culturing every 2 weeks and used for generating genome-edited plants.

### Guide RNA design and CRISPR/Cas9 plasmid construction

*CRISPR guide RNA design*: Complete genome sequence of nine badnaviruses, four of which were different BSV strains [*banana streak virus* strain Obino l’Ewai (BSOLV, GenBank accession KJ013506 and JQ409539), *banana streak VN virus* (GenBank accession KJ013510), *banana streak virus Acuminata Vietnam* (NCBI reference sequence NC_007003), *banana streak Mysore virus* (NCBI reference sequence NC_006955)] affecting banana and five other badnaviruses [*rubus yellow net virus* (GenBank accession KF241951), *gooseberry vein banding-associated virus* (GenBank accession HQ852248), *grapevine vein-clearing virus* (NCBI reference sequence NC_015784), *piper yellow mottle virus* (GenBank accession KC808712), *taro bacilliform virus* (NCBI reference sequence NC_004450)] affecting different plant species were downloaded from NCBI. The sequences were aligned to identify conserved regions related to the BSOLV, which is widely distributed in Africa. A relative conserved block of sequence was identified within each ORF of BSOLV strain. The gRNAs were designed for each of CRISPR target from sequences of the BSOLV (GenBank accession KJ013506 and JQ409539) and eBSOLV (GenBank accession HE983609 and AP009334). One gRNA was selected from each ORF (gRNA1-TTGAGCAAAGAAGACGTTG targeting S1 in ORF1; gRNA2-GCTGGAACAACTGGTGACT targeting S2 in ORF2; gRNA3-ATGGAGTTCATATGATCAT targeting S3 in ORF3) based on their specificity to their target site and minimal potential off targets in the *Musa* genome.

*CRISPR/Cas9 plasmid preparation*: The gRNA cassette, containing OsU6 promoter followed by two *BbsI* restriction sites and tracer RNA scaffold, was amplified from pZK_OsU6-gRNA plasmid^34^ using primers OsU6_Chimera.F1 and OsU6_Chimera.R1 and was cloned into pENTR-D/Topo. The additional *BbsI* site in pENTR-D/Topo backbone was mutated by amplifying with primers noBbsI.For and noBbsI.Rev (Supplementary Table 1) and self-circularizing using In-Fusion (Takara Bio USA Inc.) according to manufacturer’s instructions to generate pMR185.

One gRNA (Supplementary Table 1) from each ORF of the BSV genomic sequence was synthesized and cloned into pMR185 to generate the gRNA modules as described^8^. Briefly, for each gRNA sequence, two complementary oligos with 4 bp overhangs were annealed and inserted into *BbsI* digested pMR185 through T4 ligation.

To allow combining three gRNA modules from the three individual clones (one for each ORF), primers were designed (Supplementary Table 1) to amplify the gRNA modules adding appropriate *attB*/*attBr* flanking sites. The amplified fragments were then cloned into the appropriate Multisite Gateway Pro3 pDONR221 vectors by Gateway BP reaction to generate entry clones suitable for MultiSite Gateway LR cloning. Then, all the three gRNAs were combined into one destination vector pDe-CAS9-Kan^8^ using Gateway LR II Plus clonase reaction (ThermoFisher) to yield the final plasmid pMR215-BSV1. The plasmid has *npt*II gene as an *in planta* selection marker and each gRNA was driven by the rice Pol III promoter (OsU6 promoter). The *Cas9* gene used in this plasmid was Arabidopsis codon optimized and regulated by parsley ubiquitin promoter (PcUbi).

### Delivery of CRISPR/Cas9 plasmid into plantain

The CRISPR/Cas9 construct was introduced into *A. tumefaciens* strain EHA105 through electroporation. *Agrobacterium*-mediated transformation of embryogenic cells of Gonja Manjaya was performed with the plasmid construct using the method as described^32^. The embryogenic cell suspensions of Gonja Manjaya were cocultivated with *A. tumefaciens* strain EHA105 harboring CRISPR/Cas9 plasmid construct for 3 days in the dark at 22 °C. After 3 days, *Agrobacterium*-infected cell suspensions was washed with liquid callus induction medium supplemented with 500 mg L^−1^ cefotaxime, and transferred to selective regeneration medium supplemented with 300 mg L^−1^ cefotaxime and 100 mg L^−1^ kanamycin with fortnightly transfer onto fresh medium.

Transformed events regenerated on selective medium containing kanamycin (100 mg L^−1^) were maintained by routine sub-culturing and multiplied on proliferation medium at 26‒28 °C and a photoperiod of 16 h/8 h (day/night).

### PCR analysis to confirm integration of *Cas9* gene

Genomic DNA was extracted from putative transgenic events and control non-transgenic plants using the CTAB method as described above. The presence of *Cas9* gene was checked by PCR analysis using primers P5 (Supplementary Table 1) amplifying a region between *Cas9* and Pea 3 A poly A. PCR was performed in a 20 µL reaction volume containing 1 µL genomic DNA (100 ng µL^−1^), 2 µL of 10X PCR buffer, 0.4 µL of dNTP, 0.5 µL of 10 µM of each primer, 0.1 µL *Taq* polymerase, and 15.5 µL nuclease free water. PCR amplification conditions were performed as follows: initial denaturation step at 95 °C for 5 min, followed by 34 cycles of denaturation at 94 °C for 30 s, annealing at 55 °C for 30 s, extension at 72 °C for 1 min, and final extension at 72 °C for 10 min. After amplification, 10 µL of PCR product was resolved on 1% agarose gel stained with gel red.

### Molecular analysis of transgenic events

*PCR band shift analysis***:** The mutations in the transgenic events were confirmed by PCR analysis using P1 primer pair (Supplementary Table 1) flanking gRNAs from ORF1 and ORF2 of BSOLV. The primer pair P1 was designed to amplify a DNA fragment of 786 bp surrounding the target sites S1 and S2 (Fig. 1). PCR reaction conditions were: initial denaturation step at 95 °C for 15 min, followed by 34 cycles of denaturation at 94 °C for 30 s, annealing at 57 °C for 30 s, extension at 72 °C for 1 min, and final extension at 72 °C for 10 min. PCR amplicons were resolved on 1% agarose gel stained with gel red and observed for the shift in band size due to the deletions in the target sites.

*T7E1 assay*: For the T7E1 assay, the DNA fragments containing the targeted sites for ORF1 and ORF2 were amplified using the primer P1 (Fig. 1, Supplementary Table 1) and Phusion high-fidelity DNA polymerase (NEB). The PCR product was then denatured-annealed at 95 °C for 5 min, ramped down to 25 °C at 0.1 °C s^−1^, and incubated at 25 °C for another 30 min. Annealed PCR products were then digested with 5U of T7E1 for 2 h at 37 °C. The T7E1-digested products were separated by 1% agarose gel electrophoresis and stained with gel red.

*Sequence analysis*: PCR product obtained using BSOLV-specific primers P1 and P3 flanking target sites were purified with QIAquick PCR purification Kit (Qiagen) according to the instruction manual. The purified products were cloned to pCR*™*8*/GW/*TOPO^®^ (Invitrogen) according to the manufacturer’s instruction, transformed to DH5α chemical competent *E. coli* cells and selected on LB plates containing spectinomycin. Ten clones from each transgenic event were prepared for Sanger sequencing. A total of 20 transgenic events along with four different wild type control plants were sequenced. The sequencing data were analyzed using SnapGene software (www.snapgene.com). All the sequences were aligned with the reference sequence of the BSOLV and wild type non-edited control plant (Supplementary Notes 1 and 2). The wild type control plants were developed along with the genome-edited plants from the same embryogenic cell suspension line.

### Analysis of potential off-target mutations

The CRISPR web tool Breaking-Cas^35^ (http://bioinfogp.cnb.csic.es/tools/breakingcas) was used to check for the potential off target sequences. In addition, a 22 bp-long sequence of each gRNA including the PAM motif was blasted against banana genome A (*Musa acuminata*) and genome B (*M. balbisiana*) using the BLASTN program in the Banana Genome Hub^36^ (http://banana-genome-hub.southgreen.fr) to identify the genes with potential off-target sites. Based on the gene sequences, primers were designed to analyze the potential off-target fragments from seven potential off-target sites, which either showed high sequence similarity with the gRNAs and/or contain the PAM motif. PCR was performed with nine edited events (1, 17, 48, 66, 76, 81, 85, 87, and 97) using these primers. PCR products were purified and cloned to *pCR*™*8*/GW/*TOPO*^®^ (Invitrogen) for sequencing. The sequence chromatograms were analyzed with SnapGene software (www.snapgene.com) and compared with the banana genome and wild type non-edited control plant (Supplementary Note 3).

### Evaluation of plants for induction of BSV symptoms

*Glasshouse assay under stress conditions*: Eight randomly selected genome-edited events (17, 48, 66, 76, 81, 85, 87, and 97) confirmed by sequencing for mutations, and three control wild type plants were acclimatized and grown in pots in the greenhouse. Three replicates per event were used in this assay. Two-month-old potted plants were subjected to water stress for 2 weeks to induce BSV symptoms. At the end of the stress period, the plants were assessed visually for the presence or absence of BSV disease symptoms as chlorosis or yellow streaks. The plants were monitored for 60 days after induction of water stress. The plants were observed for BSV disease symptoms as yellowish streaks and distorted shapes of banana leaves and more severe symptoms including retarded growth, pseudostem split, and leaf necrosis leading to death of infected plants^3^.

*PCR and qPCR analysis for detection of BSOLV***:** Genomic DNA was extracted from symptomatic (66 and 97) and asymptomatic (17, 48, 76, 81, 85, and 87) edited plants and symptomatic wild type control plants using CTAB^33^. The leaves showing BSV symptoms from three replicates for each event and three control plants were pooled for DNA extraction. The PCR was performed using P4 primer pair as described above. Primer P4 can detect only the episomal BSOLV and not eBSOLV^7^. The symptomatic field plants of Agbagba (AAB) and symptomatic wild type control from glasshouse showing BSV symptoms under stress conditions were used as positive controls.

The symptomatic (66 and 97) and asymptomatic (17, 48, 76, 81, 85, and 87) edited plants and symptomatic wild type non-edited control plants from glasshouse were further validated by qPCR for detection of episomal BSOLV using P4 primers. DNA from symptomatic field plant of Agbagba plant and asymptomatic in vitro plantlet of Cavendish Williams were used as positive and negative controls, respectively. qPCR was performed in a 20 µL reaction volume containing 60 ng of genomic DNA, 10 µL of Luna Universal qPCR master mix (New England Biolab), and 0.3 µL of 10 µM of each primer (P4F and P4R, Supplementary Table 1). qPCR was performed using the ABI 7500 real-time machine (Applied Biosystem, USA). Three technical replicates were used for each sample and the experiment was performed twice. The means and standard deviation were calculated for CT values of six technical replicates from two independent experiments, using Minitab 14 statistical software, 2012. Mean CT values of genome-edited plants were compared with wild type non-edited control plant, the positive control, and the negative control.

## Supplementary information

Description of Supplementary Data

Supplementary Information

Supplementary Data 1

## Data Availability

All data needed to evaluate the conclusions in the paper are present in the paper and/or the supplementary materials. All relevant data are also available from the authors.
