# Supplementary material for: CRISPR/Cas9 editing of endogenous banana streak virus in the B genome of Musa spp. overcomes a major challenge in banana breeding
Source: Commun Biol. 2019 Jan 31;2:46. doi: 10.1038/s42003-019-0288-7 (PMC6355771; doi:10.1038/s42003-019-0288-7)
Supplement: Supplementary file 1 — Description of Supplementary Data [file 42003_2019_288_MOESM1_ESM.docx]

**Description of Additional Supplementary Files**

**File Name**: Supplementary Data 1

**Description**: Source data used for making figure 5c
