# Supplementary material for: CRISPR/Cas9 editing of endogenous banana streak virus in the B genome of Musa spp. overcomes a major challenge in banana breeding
Source: Commun Biol. 2019 Jan 31;2:46. doi: 10.1038/s42003-019-0288-7 (PMC6355771; doi:10.1038/s42003-019-0288-7)
Supplement: Supplementary file 2 — Supplementary Information [file 42003_2019_288_MOESM2_ESM.pdf]

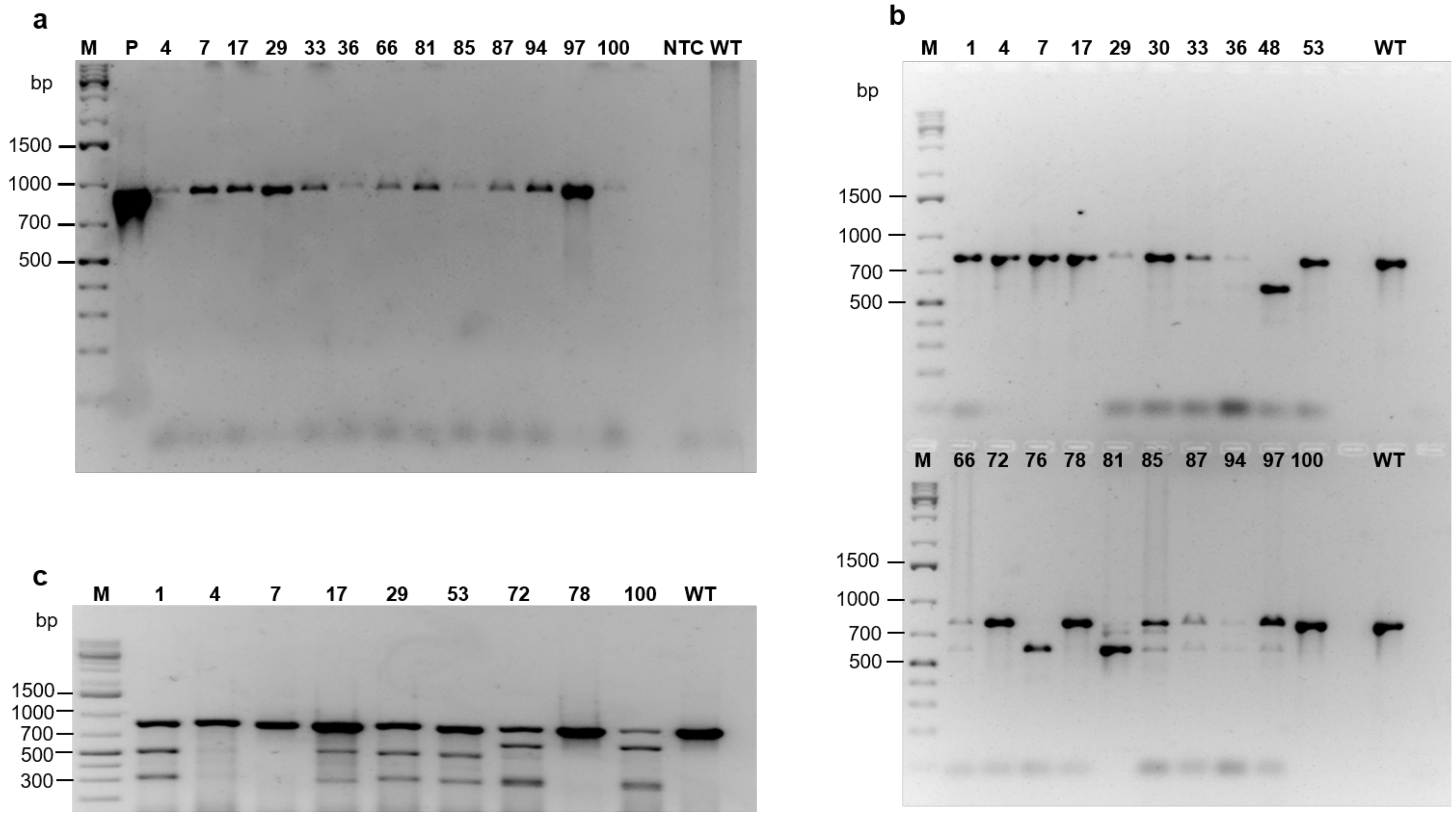

Supplementary Figure 1: Molecular characterization of transgenic events of Gonja Manjaya. **a)** PCR amplification to confirm the presence of *Cas9* gene in transgenic events. **b)** Band shift analysis of genomic DNA of transgenic events by PCR amplification using primer set P1 to detect mutations at target sites S1 and S2. If Cas9 cleaves the two gRNAs simultaneously, mutant plants will have band shift of 197 bp compared to the wild type non-edited control plant. **c)** T7 endonuclease I assay to detect mutations in the transgenic plants. The DNA fragments were amplified by PCR using primer set P1 and mismatches resulting from deletion or insertion at S1 and S2, were detected by T7 endonuclease I digestion. 1-100, independent transgenic events; WT, wild type non-transgenic control plant; P, plasmid pMR215-BSV1 as positive control; M, molecular marker.

Supplementary Table 1: List of primers used in the study.

Primer	Sequence 5'-3'
gRNA1 (S1 in ORF1)	S1F GTTGTTGAGCAAAGAAGACGTTG (underlined nucleotides are adapters)<br>S1R AAACCAACGTCTTCTTTGCTCAA
gRNA2 (S2 in ORF2)	S2F GTTGGCTGGAACAACTGGTGACT (underlined nucleotides are adapters)<br>S2R AAACAGTCACCAGTTGTTCCAGC
gRNA3 (S3 in ORF3)	S3F GTTGATG GAGTTCATATGATCAT (underlined nucleotides are adapters)<br>S3R AAACATGATCATATGAACTCCAT
OsU6_chimera.F1	CACCGGATCATGAACCAACGGCCTGGCTG
OsU6_chimera.R1	GGGAATTGCCCTTCGAAGGGAC
noBbsI.For	AGTCTTGCGACTGAGCCTTTCGTTTTATTTGATGCC
noBbsI.Rev	CTCAGTCGCAAGACTGGGCCTTTCGTTTTATCTG
P1F	CAACGGAAGCCAAAGTCTCA
P1R	CCACCGTCTTTCCTTGGTTG
P2F	TGGAAGGGTTGTAGGACGAC
P2R	TGCTTCACCTTGGAATCCCT
P3F	TGGGTTGGTAAGCAATGGTTC
P3R	GCTTCTGGACATGCTTAGTCA
P4F	TTGGTGTTTAACTATAAGAGGCTGAA
P4R	TTATTGCATCCACATTTGAAAAC
P5F	GGATCTCATCATCAAGCTCCCAA
P5R	TGCACCTTAATATCACACTGTTAGTTCC
Ma06_t37830.1F	GTGAATGGACGAGGCAAGAC
Ma06_t37830.1R	TACCATGCAGAATTGGGAAC
Ma05_t06600.1F	TGCCCACAATTGATAGGTGA
Ma05_t06600.1R	GCATATAAGCCCGACCGTAA
Ma01_t31620.1F	GCCTTGGCCATGCAGACACA

Ma01_t31620.1R	GTCAAGACTTACAGCTTACG
Ma01_t10610.1F	ACCAGGGCACGTGCCTCACAA
Ma01_t10610.1R	GACACACACATCAAACTTCTTTTG
Ma08_t27880.1F	GTTTTGGGTTTGCTTGAACC
Ma08_t27880.1R	TCACAAGCATGGATGTCGTT
Bchr6_T18444F	ATGGACGAGGCAAGACGTGGA
Bchr6_T18444R	TGGATCCTCACTGTCGGCGT
Bchr5_T12273F	AAAATTGGAGCCATCAGCAC
Bchr5_T12273R	AGCCAAGCTATCCTGTGGAA
Bchr4_T08704F	TGCTCATGACCCATTCATCA
Bchr4_T08704R	TATAGGCCTGCTCGATTCCA
Bchr1_T00896F	AGCAATCTCACGAAGGAGGA
Bchr1_T00896R	GCAGCTTCAACTTCAGTGTCG
pDONR_P4rP3r<br>_gRNA1	attB4r_OsU6gRNA GGGGACAACTTTTCTATACAAAGTTGTAGGATCATGAACCAACGGCCTGGCTG<br>attB3r_OsU6gRNA GGGGACAACTTTATTATACAAAGTTGTAATTGCCCTTCGAAGGGACAAAAAAAGC
pDONR_P1P4_<br>gRNA2	attB1_OsU6gRNA GGGGACAAGTTTGTACAAAAAAGCAGGCTTAGGATCATGAACCAACGGCCTGGCTG<br>attB4_OsU6gRNA GGGGACAACTTTGTATAGAAAAGTTGGGTGAATTGCCCTTCGAAGGGACAAAAAAAGC
pDONR_P3P2_<br>gRNA3	attB3_OsU6gRNA GGGGACAACTTTGTATAATAAAGTTGTAGGATCATGAACCAACGGCCTGGCTG<br>attB2_OsU6gRNA GGGGACCACTTTGTACAAGAAAGCTGGGTTAATTGCCCTTCGAAGGGACAAAAAAAGC

Supplementary Table 2: List of potential off-target sites for mutation identified in A and B genome of banana.

<table>
<tr><th rowspan="2">gRNA sequence from BSV + PAM</th><th colspan="8">Potential off target sites in A genome (Musa acuminata)</th></tr>
<tr><th>Gene ID</th><th>Start nucleotide</th><th>End nucleotide</th><th>Chromosome number</th><th>Start hit</th><th>End hit</th><th>Identity (%)</th><th>Sequence alignment</th></tr>
<tr><td rowspan="12">TTGAGCAAAGAAGACGTTGAGG</td><td></td><td>1</td><td>20</td><td>Chr07</td><td>2863198</td><td>2863217</td><td>86.36</td><td>TTGAGCAAAGAAGACGTTGA
|| || ||||||||||||||
TTAAGGAAAGAAGACGTTGA</td></tr>
<tr><td>Ma06_t33580.1</td><td>3</td><td>20</td><td>Chr06</td><td>34157611</td><td>34157628</td><td>77.27</td><td>GAGCAAAGAAGACGTTGA
|||||||||||| |||||
GAGCAAAGAAGAAGTTGA</td></tr>
<tr><td>Ma05_t06600.1</td><td>3</td><td>20</td><td>Chr05</td><td>4885359</td><td>4885376</td><td>77.27</td><td>GAGCAAAGAAGACGTTGA
|||||||||||| |||||
GAGCAAAGAAGAAGTTGA</td></tr>
<tr><td>Ma09_t30220.1</td><td>4</td><td>19</td><td>Chr09</td><td>40456491</td><td>40456506</td><td>68.18</td><td>AGCAAAGAAGACGTTG
||||||||||||||||
AGCAAAGAAGACGTTG</td></tr>
<tr><td>Ma06_t37830.1</td><td>8</td><td>22</td><td>Chr06</td><td>36778699</td><td>36778713</td><td>63.64</td><td>AAGAAGACGTTGAGG
|||||||||||||||
AAGAAGACGTTGAGG</td></tr>
<tr><td>Ma09_t25390.1</td><td>5</td><td>19</td><td>Chr09</td><td>36870057</td><td>36870071</td><td>63.64</td><td>GCAAAGAAGACGTTG
|||||||||||||||
GCAAAGAAGACGTTG</td></tr>
<tr><td></td><td>2</td><td>16</td><td>Chr05</td><td>21277120</td><td>21277134</td><td>63.64</td><td>TGAGCAAAGAAGACG
|||||||||||||||
TGAGCAAAGAAGACG</td></tr>
<tr><td></td><td>1</td><td>15</td><td>Chr05</td><td>1352096</td><td>1352110</td><td>63.64</td><td>TTGAGCAAAGAAGAC
|||||||||||||||
TTGAGCAAAGAAGAC</td></tr>
<tr><td>Ma05_t09410.1</td><td>6</td><td>20</td><td>Chr05</td><td>6800092</td><td>6800106</td><td>63.64</td><td>CAAAGAAGACGTTGA
|||||||||||||||
CAAAGAAGACGTTGA</td></tr>
<tr><td>Ma11_t18440.1</td><td>2</td><td>16</td><td>Chr11</td><td>23494840</td><td>23494854</td><td>63.64</td><td>TGAGCAAAGAAGACG
|||||||||||||||
TGAGCAAAGAAGACG</td></tr>
<tr><td>Ma07_t07430.1</td><td>1</td><td>15</td><td>Chr07</td><td>5522379</td><td>5522393</td><td>63.64</td><td>TTGAGCAAAGAAGAC
|||||||||||||||
TTGAGCAAAGAAGAC</td></tr>
</table>

<table>
<tr><td rowspan="13">GCTGGAACAACTGGTGACTGGG</td><td>Ma05_t31620.1</td><td>1</td><td>23</td><td>Chr05</td><td>41456271</td><td>41456293</td><td>95.65</td><td>TGCTGGAACAACTGGTGACTGGG
||||| || | ||||||||||||
TGCTGAAAAATCTGGTGACTGGG</td></tr>
<tr><td>Ma01_t10610.1</td><td>2</td><td>21</td><td>Chr01</td><td>7639726</td><td>7639745</td><td>86.36</td><td>CTGGAACAACTGGTGACTGG
|||| ||||||||||||||
CTGGTCCAACTGGTGACTGG</td></tr>
<tr><td>Ma09_t17510.1</td><td>3</td><td>21</td><td>Chr09</td><td>13077000</td><td>13077017</td><td>77.27</td><td>TGGAACAACTGGTGACTG
||||||||||| ||||||
TGGAACAACTGATGACTG</td></tr>
<tr><td>Ma04_t03550.1</td><td>1</td><td>15</td><td>Chr04</td><td>2727599</td><td>2727613</td><td>63.64</td><td>GCTGGAACAACTGGT
|||||||||||||||
GCTGGAACAACTGGT</td></tr>
<tr><td>Ma04_t18600.1</td><td>3</td><td>17</td><td>Chr04</td><td>20644374</td><td>20644388</td><td>63.64</td><td>TGGAACAACTGGTGA
|||||||||||||||
TGGAACAACTGGTGA</td></tr>
<tr><td>Ma04_t03410.2</td><td>7</td><td>21</td><td>Chr04</td><td>2637520</td><td>2637534</td><td>63.64</td><td>ACAACTGGTGACTGG
|||||||||||||||
ACAACTGGTGACTGG</td></tr>
<tr><td></td><td>3</td><td>17</td><td>Chr03</td><td>15645024</td><td>15645038</td><td>63.64</td><td>TGGAACAACTGGTGA
|||||||||||||||
TGGAACAACTGGTGA</td></tr>
<tr><td>Ma05_t01980.1</td><td>6</td><td>20</td><td>Chr05</td><td>1247491</td><td>1247505</td><td>63.64</td><td>AACAACTGGTGACTG
|||||||||||||||
AACAACTGGTGACTG</td></tr>
<tr><td>Ma11_t06300.1</td><td>7</td><td>21</td><td>Chr11</td><td>5090027</td><td>5090041</td><td>63.64</td><td>ACAACTGGTGACTGG
|||||||||||||||
ACAACTGGTGACTGG</td></tr>
<tr><td></td><td>6</td><td>20</td><td>Chr07</td><td>12776568</td><td>12776582</td><td>63.64</td><td>AACAACTGGTGACTG
|||||||||||||||
AACAACTGGTGACTG</td></tr>
<tr><td>Ma08_t28040.1</td><td>1</td><td>15</td><td>Chr08</td><td>40099833</td><td>40099847</td><td>63.64</td><td>GCTGGAACAACTGGT
|||||||||||||||
GCTGGAACAACTGGT</td></tr>
<tr><td></td><td>2</td><td>16</td><td>Chr08</td><td>11139839</td><td>11139853</td><td>63.64</td><td>CTGGAACAACTGGTG
|||||||||||||||
CTGGAACAACTGGTG</td></tr>
<tr><td>Ma10_t05740.1</td><td>1</td><td>15</td><td>Chr10</td><td>17114823</td><td>17114837</td><td>63.64</td><td>GCTGGAACAACTGGT
|||||||||||||||
GCTGGAACAACTGGT</td></tr>
</table>

<table>
<tr><td rowspan="13">ATGGAGTTCATATGATCATAGG</td><td>Ma08_t27880.1</td><td>1</td><td>20</td><td>Chr08</td><td>40001548</td><td>40001567</td><td>86.36</td><td>ATGGAGTTCATATGATCATA
|||||| ||||||||||||
ATGGAGACCATATGATCATA</td></tr>
<tr><td></td><td>1</td><td>20</td><td>Chr03</td><td>25523263</td><td>25523282</td><td>86.36</td><td>ATGGAGTTCATATGATCATA
||| |||||||||||| |||
ATGTAGTTCATATGATTATA</td></tr>
<tr><td>Ma01_t00530.1</td><td>2</td><td>21</td><td>Chr01</td><td>385460</td><td>385479</td><td>86.36</td><td>TGGAGTTCATATGATCATAG
||||||||||||| | ||||
TGGAGTTCATATGTTTATAG</td></tr>
<tr><td></td><td>2</td><td>20</td><td>Chr08</td><td>32038771</td><td>32038789</td><td>81.82</td><td>TGGAGTTCATATGATCATA
||| |||||||||||||||
TGGTGTTCATATGATCATA</td></tr>
<tr><td></td><td>2</td><td>20</td><td>Chr10</td><td>7743722</td><td>7743740</td><td>81.82</td><td>TGGAGTTCATATGATCATA
||| |||||||||||||||
TGGTGTTCATATGATCATA</td></tr>
<tr><td>Ma09_t21950.1</td><td>1</td><td>18</td><td>Chr09</td><td>33919002</td><td>33919019</td><td>77.27</td><td>ATGGAGTTCATATGATCA
||||||||||||| ||||
ATGGAGTTCATATCATCA</td></tr>
<tr><td></td><td>2</td><td>19</td><td>Chr09</td><td>27358998</td><td>27359015</td><td>77.27</td><td>TGGAGTTCATATGATCAT
|||| |||||||||||||
TGGAATTCATATGATCAT</td></tr>
<tr><td>Ma01_t10050.1</td><td>4</td><td>20</td><td>Chr01</td><td>7190459</td><td>7190475</td><td>72.73</td><td>GAGTTCATATGATCATA
|||||||||||||||||
GAGTTCATATGATCATA</td></tr>
<tr><td>Ma04_t11810.1</td><td>6</td><td>21</td><td>Chr04</td><td>8419515</td><td>8419530</td><td>68.18</td><td>GTTCATATGATCATAG
||||||||||||||||
GTTCATATGATCATAG</td></tr>
<tr><td>Ma04_t21240.1</td><td>4</td><td>19</td><td>Chr04</td><td>23795046</td><td>23795061</td><td>68.18</td><td>GAGTTCATATGATCAT
||||||||||||||||
GAGTTCATATGATCAT</td></tr>
<tr><td>Ma05_t28500.1</td><td>4</td><td>19</td><td>Chr05</td><td>39488377</td><td>39488392</td><td>68.18</td><td>GAGTTCATATGATCAT
||||||||||||||||
GAGTTCATATGATCAT</td></tr>
<tr><td>Ma05_t18370.1</td><td>2</td><td>17</td><td>Chr05</td><td>23654494</td><td>23654509</td><td>68.18</td><td>TGGAGTTCATATGATC
||||||||||||||||
TGGAGTTCATATGATC</td></tr>
<tr><td></td><td>6</td><td>20</td><td>ChrUn_random</td><td>42307679</td><td>42307693</td><td>63.64</td><td>GTTCATATGATCATA
|||||||||||||||
GTTCATATGATCATA</td></tr>
</table>

<table>
<tr><td></td><td></td><td>8</td><td>22</td><td>Chr04</td><td>21341215</td><td>21341229</td><td>63.64</td><td>TCATATGATCATAGG
| | | | | | | | | | | | | | |
TCATATGATCATAGG</td></tr>
<tr><td></td><td></td><td>1</td><td>15</td><td>Chr04</td><td>33115415</td><td>33115429</td><td>63.64</td><td>ATGGAGTTCATATGA
| | | | | | | | | | | | | | |
ATGGAGTTCATATGA</td></tr>
<tr><td></td><td>Ma05_t25660.1</td><td>7</td><td>21</td><td>Chr05</td><td>37522305</td><td>37522319</td><td>63.64</td><td>TTCATATGATCATAG
| | | | | | | | | | | | | | |
TTCATATGATCATAG</td></tr>
<tr><td></td><td></td><td>6</td><td>20</td><td>Chr05</td><td>22429911</td><td>22429925</td><td>63.64</td><td>GTTCATATGATCATA
| | | | | | | | | | | | | | |
GTTCATATGATCATA</td></tr>
<tr><td></td><td></td><td>6</td><td>20</td><td>Chr05</td><td>1135757</td><td>1135771</td><td>63.64</td><td>GTTCATATGATCATA
| | | | | | | | | | | | | | |
GTTCATATGATCATA</td></tr>
<tr><td></td><td></td><td>3</td><td>17</td><td>Chr06</td><td>29511504</td><td>29511518</td><td>63.64</td><td>GGAGTTCATATGATC
| | | | | | | | | | | | | | |
GGAGTTCATATGATC</td></tr>
<tr><td></td><td></td><td>6</td><td>20</td><td>Chr06</td><td>16476946</td><td>16476960</td><td>63.64</td><td>GTTCATATGATCATA
| | | | | | | | | | | | | | |
GTTCATATGATCATA</td></tr>
<tr><td></td><td></td><td>3</td><td>17</td><td>Chr06</td><td>9077312</td><td>9077326</td><td>63.64</td><td>GGAGTTCATATGATC
| | | | | | | | | | | | | | |
GGAGTTCATATGATC</td></tr>
<tr><td></td><td></td><td>6</td><td>20</td><td>Chr06</td><td>18270895</td><td>18270909</td><td>63.64</td><td>GTTCATATGATCATA
| | | | | | | | | | | | | | |
GTTCATATGATCATA</td></tr>
<tr><td></td><td>Ma03_t30210.1</td><td>7</td><td>21</td><td>Chr03</td><td>32945445</td><td>32945459</td><td>63.64</td><td>TTCATATGATCATAG
| | | | | | | | | | | | | | |
TTCATATGATCATAG</td></tr>
<tr><td></td><td></td><td>5</td><td>19</td><td>Chr03</td><td>7232551</td><td>7232565</td><td>63.64</td><td>AGTTCATATGATCAT
| | | | | | | | | | | | | | |
AGTTCATATGATCAT</td></tr>
<tr><td></td><td></td><td>5</td><td>19</td><td>Chr03</td><td>7235003</td><td>7235017</td><td>63.64</td><td>AGTTCATATGATCAT
| | | | | | | | | | | | | | |
AGTTCATATGATCAT</td></tr>
<tr><td></td><td></td><td>6</td><td>20</td><td>Chr03</td><td>29098688</td><td>29098702</td><td>63.64</td><td>GTTCATATGATCATA
| | | | | | | | | | | | | | |
GTTCATATGATCATA</td></tr>
</table>

<table>
<tr><td></td><td>Ma11_t12150.1</td><td>1</td><td>15</td><td>Chr11</td><td>15988683</td><td>15988697</td><td>63.64</td><td>ATGGAGTTCATATGA
|||||||||||||||
ATGGAGTTCATATGA</td></tr>
<tr><td></td><td></td><td>6</td><td>20</td><td>Chr07</td><td>22829650</td><td>22829664</td><td>63.64</td><td>GTTCATATGATCATA
|||||||||||||||
GTTCATATGATCATA</td></tr>
<tr><td></td><td>Ma07_t17510.1</td><td>5</td><td>19</td><td>Chr07</td><td>20226213</td><td>20226227</td><td>63.64</td><td>AGTTCATATGATCAT
|||||||||||||||
AGTTCATATGATCAT</td></tr>
<tr><td></td><td>Ma07_t18860.1</td><td>5</td><td>19</td><td>Chr07</td><td>26790705</td><td>26790719</td><td>63.64</td><td>AGTTCATATGATCAT
|||||||||||||||
AGTTCATATGATCAT</td></tr>
<tr><td></td><td></td><td>6</td><td>20</td><td>Chr07</td><td>20165904</td><td>20165918</td><td>63.64</td><td>GTTCATATGATCATA
|||||||||||||||
GTTCATATGATCATA</td></tr>
<tr><td></td><td></td><td>6</td><td>20</td><td>Chr10</td><td>15677881</td><td>15677895</td><td>63.64</td><td>GTTCATATGATCATA
|||||||||||||||
GTTCATATGATCATA</td></tr>
<tr><td></td><td></td><td>6</td><td>20</td><td>Chr02</td><td>23388132</td><td>23388146</td><td>63.64</td><td>GTTCATATGATCATA
|||||||||||||||
GTTCATATGATCATA</td></tr>
<tr><td></td><td></td><td>5</td><td>19</td><td>Chr02</td><td>26012267</td><td>26012281</td><td>63.64</td><td>AGTTCATATGATCAT
|||||||||||||||
AGTTCATATGATCAT</td></tr>
</table>

<table>
<tr><th rowspan="2">gRNA sequence from BSV + PAM</th><th colspan="8">Potential off target sites in B genome (Musa balbisiana)</th></tr>
<tr><th>Gene ID</th><th>Start nucleotide</th><th>End nucleotide</th><th>Chromosome number</th><th>Start hit</th><th>End hit</th><th>Identity (%)</th><th>Sequence alignment</th></tr>
<tr><td rowspan="3">TTGAGCAAAGAAGACGTTGAGG</td><td></td><td>2</td><td>21</td><td>Chr05</td><td>2521108</td><td>2521126</td><td>81.82</td><td>TGAGCAAAGAAGACGTTGAG
||||||| ||||||||||||
TGAGCAA-GAAGACGTTGAG</td></tr>
<tr><td>ITC1587_Bchr6_T18081</td><td>3</td><td>20</td><td>Chr06</td><td>24830292</td><td>24830309</td><td>77.27</td><td>GAGCAAAGAAGACGTTGA
|||||||||||| |||||
GAGCAAAGAAGAAGTTGA</td></tr>
<tr><td>ITC1587_Bchr5_T12273</td><td>3</td><td>20</td><td>Chr05</td><td>4536286</td><td>4536303</td><td>77.27</td><td>GAGCAAAGAAGACGTTGA
|||||||||||| |||||
GAGCAAAGAAGAAGTTGA</td></tr>
</table>

<table>
<tr><td></td><td></td><td>4</td><td>19</td><td>Chr09</td><td>25132571</td><td>25132586</td><td>68.18</td><td>AGCAAAGAAGACGTTG
||||||||||||||||
AGCAAAGAAGACGTTG</td></tr>
<tr><td></td><td>ITC1587_Bchr7_P19225</td><td>1</td><td>15</td><td>Chr07</td><td>5146248</td><td>5146262</td><td>63.64</td><td>TGAGCAAAGAAGAC
|||||||||||||||
TTGAGCAAAGAAGAC</td></tr>
<tr><td></td><td>ITC1587_Bchr6_T18444</td><td>8</td><td>22</td><td>Chr06</td><td>27129733</td><td>27129747</td><td>63.64</td><td>AAGAAGACGTTGAGG
|||||||||||||||
AAGAAGACGTTGAGG</td></tr>
<tr><td></td><td>ITC1587_Bchr11_T33819</td><td>2</td><td>16</td><td>Chr11</td><td>16621602</td><td>16621616</td><td>63.64</td><td>TGAGCAAAGAAGACG
|||||||||||||||
TGAGCAAAGAAGACG</td></tr>
<tr><td></td><td>ITC1587_Bchr5_T12522</td><td>6</td><td>20</td><td>Chr05</td><td>6186959</td><td>6186973</td><td>63.64</td><td>CAAAGAAGACGTTGA
|||||||||||||||
CAAAGAAGACGTTGA</td></tr>
<tr><td></td><td></td><td>2</td><td>16</td><td>Chr05</td><td>13390573</td><td>13390587</td><td>63.64</td><td>TGAGCAAAGAAGACG
|||||||||||||||
TGAGCAAAGAAGACG</td></tr>
<tr><td>GCTGGAACAACTGGTGACTGGG</td><td></td><td>3</td><td>20</td><td>Chr09</td><td>11368005</td><td>11368022</td><td>77.27</td><td>TGGAACAACTGGTGACTG
||||||||||| ||||||
TGGAACAACTGATGACTG</td></tr>
<tr><td></td><td>ITC1587_Bchr4_T08704</td><td>1</td><td>15</td><td>Chr04</td><td>2353370</td><td>2353384</td><td>63.64</td><td>GCTGGAACAACTGGT
|||||||||||||||
GCTGGAACAACTGGT</td></tr>
<tr><td></td><td></td><td>6</td><td>20</td><td>Chr07</td><td>11623702</td><td>11623716</td><td>63.64</td><td>AACAACTGGTGACTG
|||||||||||||||
AACAACTGGTGACTG</td></tr>
<tr><td></td><td></td><td>1</td><td>15</td><td>Chr10</td><td>869294</td><td>869308</td><td>63.64</td><td>GCTGGAACAACTGGT
|||||||||||||||
GCTGGAACAACTGGT</td></tr>
<tr><td></td><td>ITC1587_Bchr5_T11827</td><td>6</td><td>20</td><td>Chr05</td><td>1144142</td><td>1144156</td><td>63.64</td><td>AACAACTGGTGACTG
|||||||||||||||
AACAACTGGTGACTG</td></tr>
<tr><td></td><td>ITC1587_Bchr8_T24443</td><td>1</td><td>15</td><td>Chr08</td><td>23286778</td><td>23286792</td><td>63.64</td><td>GCTGGAACAACTGGT
|||||||||||||||
GCTGGAACAACTGGT</td></tr>
<tr><td>ATGGAGTTCATATGATCATAGG</td><td></td><td>1</td><td>21</td><td>ChrUn_random</td><td>137660281</td><td>137660301</td><td>90.91</td><td>ATGGAGTTCATATGATCATAG
||||| ||||||||||||||
ATGGATGTCATATGATCATAG</td></tr>
</table>

<table>
<tr><td></td><td></td><td>1</td><td>21</td><td>Chr1</td><td>21774466</td><td>21774486</td><td>90.91</td><td>ATGGAGTTCATATGATCATAG
||||| ||||||||||||||
ATGGATGTCATATGATCATAG</td></tr>
<tr><td></td><td>ITC1587_BchrUn_random _T36872</td><td>1</td><td>20</td><td>ChrUn_random</td><td>65857817</td><td>65857836</td><td>86.36</td><td>ATGGAGTTCATATGATCATA
|| ||| |||||||||||||
ATAGAGATCATATGATCATA</td></tr>
<tr><td></td><td></td><td>1</td><td>20</td><td>Chr07</td><td>20607845</td><td>20607864</td><td>86.36</td><td>ATGGAGTTCATATGATCATA
|| ||||||||||||| |||
ATTGAGTTCATATGATTATA</td></tr>
<tr><td></td><td></td><td>1</td><td>20</td><td>Chr08</td><td>23191148</td><td>23191167</td><td>86.36</td><td>ATGGAGTTCATATGATCATA
|||||| |||||||||||||
ATGGAGATCATATGATCAT</td></tr>
<tr><td></td><td>ITC1587_Bchr1_T00896</td><td>2</td><td>21</td><td>Chr1</td><td>6608674</td><td>6608693</td><td>86.36</td><td>TGGAGTTCATATGATCATAG
||||||||||||| | ||||
TGGAGTTCATATGTTTATAG</td></tr>
<tr><td></td><td></td><td>2</td><td>19</td><td>Chr09</td><td>13282715</td><td>13282732</td><td>77.27</td><td>TGGAGTTCATATGATCAT
|||| |||||||||||||
TGGAATTCATATGATCAT</td></tr>
<tr><td></td><td></td><td>1</td><td>18</td><td>Chr05</td><td>5216307</td><td>5216324</td><td>77.27</td><td>ATGGAGTTCATATGATCA
||||||||||||||| ||
ATGGAGTTCATATGAACA</td></tr>
<tr><td></td><td>ITC1587_Bchr4_T01462</td><td>4</td><td>20</td><td>Chr01</td><td>10982691</td><td>10982707</td><td>72.73</td><td>GAGTTCATATGATCATA
|||||||||||||||||
GAGTTCATATGATCATA</td></tr>
<tr><td></td><td></td><td>5</td><td>20</td><td>Chr01</td><td>6490709</td><td>6490724</td><td>68.18</td><td>AGTTCATATGATCATA
||||||||||||||||
AGTTCATATGATCATA</td></tr>
<tr><td></td><td>ITC1587_Bchr1_T00046</td><td>4</td><td>19</td><td>Chr01</td><td>335679</td><td>335694</td><td>68.18</td><td>GAGTTCATATGATCAT
||||||||||||||||
GAGTTCATATGATCAT</td></tr>
<tr><td></td><td></td><td>5</td><td>20</td><td>Chr05</td><td>5209637</td><td>5209652</td><td>68.18</td><td>AGTTCATATGATCATA
||||||||||||||||
AGTTCATATGATCATA</td></tr>
<tr><td></td><td>ITC1587_Bchr3_T08047</td><td>7</td><td>21</td><td>Chr03</td><td>22264915</td><td>22264929</td><td>63.64</td><td>TTCATATGATCATAG
|||||||||||||||
TTCATATGATCATAG</td></tr>
<tr><td></td><td></td><td>5</td><td>19</td><td>Chr03</td><td>6492812</td><td>6492826</td><td>63.64</td><td>AGTTCATATGATCAT
|||||||||||||||
AGTTCATATGATCAT</td></tr>
</table>

		1	15	Chr03	1031873	1031887	63.64	ATGGAGTTCATATGA<br>||||||||||||||| ATGGAGTTCATATGA
	ITC1587_BchrUn_random_T38712	5	19	ChrUn_random	113663668	113663682	63.64	AGTTCATATGATCAT<br>||||||||||||||| AGTTCATATGATCAT
		1	15	Chr04	21070625	21070639	63.64	ATGGAGTTCATATGA<br>||||||||||||||| ATGGAGTTCATATGA
	ITC1587_Bchr2_T03632	2	16	Chr02	7174143	7174157	63.64	TGGAGTTCATATGAT<br>||||||||||||||| TGGAGTTCATATGAT
	ITC1587_Bchr11_T33165	1	15	Chr11	11377101	11377115	63.64	ATGGAGTTCATATGA<br>||||||||||||||| ATGGAGTTCATATGA
		3	17	Chr06	7042940	7042954	63.64	GGAGTTCATATGATC<br>||||||||||||||| GGAGTTCATATGATC
		3	17	Chr06	20692879	20692893	63.64	GGAGTTCATATGATC<br>||||||||||||||| GGAGTTCATATGATC
	ITC1587_Bchr5_T14203	7	21	Chr05	19805136	19805150	63.64	TTCATATGATCATAG<br>||||||||||||||| TTCATATGATCATAG
	ITC1587_Bchr5_T12106	7	21	Chr05	3420839	3420853	63.64	TTCATATGATCATAG<br>||||||||||||||| TTCATATGATCATAG
	ITC1587_Bchr5_T11815	6	20	Chr05	1042809	1042823	63.64	GTTCATATGATCATA<br>||||||||||||||| GTTCATATGATCATA
		5	19	Chr05	21560834	21560848	63.64	AGTTCATATGATCAT<br>||||||||||||||| AGTTCATATGATCAT

Supplementary Note 1: Sequence of PCR product sequenced by Sanger sequencing for confirmation of mutations. This fragment was amplified from genome edited and wild type control plants using P1 primers as underlined, covering target sites S1 and S2 in ORF1 and ORF2 of BSOLV. PAM and gRNA are represented as blue and black bold respectively.

>Target sites S1 and S2 in ORF1-ORF2 of BSOLV

CAACGGAAGCCAAAGTCTCAAACAAAGATCTTGCTCATAATTTGCACATCAACACTTATCGACTAAGCCTTACAGGGAAGGTCCTTTGGACTTCTGGAAGGA
AAAATCGGGATTTACTTGTGCAGATAATCGGTGAGCAGGAGGCTCAAAAGAAGGACTTATTAGAGTTGCAGAACTTAAGTAAGATTGTGCGCAGCCAGCGT
AACGATCTTAAGAAGGCTCACGAAAAGCTGGACGTCTTGTCTGATGAGCTTAAAGCTCTCAGAAAGGACTATCTGAAGAGGCGCCCC**TTGAGCAAAGAAG**
ACGTTGAGGAATTAGTTTTGCGTATCTCTGAACAACCTAAGTTCATAGAGAAGCAAACTGAGGCCTTAACTGAGGAGCTGACTAAAGAAGTTCAAAATCTG
AAAAAAATTATTCATGACTTTGAGCGAAAGCTCATGGGATGAGTAATTCTATCACAAGCTCTGCAGTATACCAACAAGCAATT**GCTGGAACAACTGGTGA**
CTGGGAATCCCCTGGAGTAGGGATATCTGACCGTGGATCGGTGAACAACACCCAGCTCACAAGACAACTCAACACCATTATATTCCTGTGTACAAAGACAC
AACAGGAAGTCTTAGCCTTAAAAGACACAGTAGCGGACATCCAAAACTGCTTGAGAATACTTGAAAGGACCGGTGCAACATCAGCCGGTACCCCACAACTT
AAGGGTGAAATTGACGCCATCAACGAGAAGTTATCAAGAATTCAACAAATTCAAGGGAGTCAACCAAGGAAAGACGGTGG

Supplementary Note 2: Sequence of PCR product sequenced by Sanger sequencing for confirmation of mutations. This fragment was amplified from genome edited and wild type control plants using P3 primers as underlined, covering target sites S3 in ORF3 of BSOLV. PAM and gRNA are represented as blue and black bold respectively.

> Target sites S3 in ORF3 of BSOLV

CAATTCTTGACACTGGAGCTACAGTCTGTGTAGCAGATGAGAGGATGATACCTTCAGGAATGAAAGAGCAGGCCAAAAATAAAATCATTATTCGAGGAGTC
AACGGAGTCACTGAAGTAAACGAGGTGACATCAGCGGGAAAGCTATGGGTTGGTAAGCAATGGTTCTACCTCCCTCAAACTTTTATTATGCCTTCATTAGCT
G**ATGGAGTTCATATGATCAT**AGGCATGAATTTTATTAGAACTGTTGGCCTAAGGATAGAAAATGGTGAGGTCACAATTTATAAGATCATGACAACAGTAC
AAGCCCCACCAATAGTTCATGAGCTGAATTATATTGATGAACTAGAACTGGAACTTCATGAATACTATAACATATGTGCAGCTGAGAGTTCTAGAGGGGAA
ATTTCTGAAGAATTTATATCTCCTGACATTATTGGAAAAATGAAAAAATTGGGATATATTGGAGAAGAACCTCTCAAACATTGGGAGAAAAATCAGGTGAA
ATGTAGGATTGAAGTAAAAAACCCTGATATGATTATTGAAGATAGGCCATTAAAACATGTTACCCCTACAATGAAAGAAACCATGACTAAGCATGTCCAGA
AGC

Supplementary Note 3: Sequence of potential off-target sites sequenced by Sanger sequencing for confirmation of mutations. The potential off target sites were amplified from genome edited and wild type control plants using primers as underlined. Black bold and blue indicate potential site matching with gRNA and PAM.

>Ma05_t06600.1

TGCCCACAATTGATAGGTGATTCGAAGGATTTGCTACTTTTGATAACCTGCGTTGCCCTCCCGACATCCACTGGCCGTAGATTTTTAGTTCGAGGAAGAAAAT
TGGAGCCATCAGCACGTACCATAGAGCGGCATGCAGATTGACGGCAACCGAGGGTGGCATACCGATTGCTAAGAAGAGGCAGGCTATCCATG**GAGCAAAG
AAGAAGTTGA**CTCGGATGGGGTGATAGTACTCCCGGCGGATTGCTTCAAAGTAAAATATGATCTTCAGCACGTAGATGGCGGACACAATGCCCATCAATGC
AAGTGAGATGCACCAGAGAACGAGATTTACGGTCGGGCTTATATGC

>Ma01_t31620.1

GCCTTGGCCATGCAGACACAGTAGCCCATCACTTCGAATGCTGCTCTCCTTTCGTACTGGAGAAATTTCCATCCAGCTAGGACTAGATTGACTGTTAACCAAA
AGGAAATGAACCATATCCTCTTCCAGTTCTCATGAACAAAATCTGTGGTCA**TGCTGAAAAATCTGGTGACTGGG**TTTCTGTATTTCAGCGGTATCATTGTTC
TCGCAAGGCTCTGCGAACTTTTTGTTAACTCGGCTCCGTCTGAGCTAACCATCCCTTGAAGTAATGTCTCAAGCTGCCAAAGCTGGAACAGAGAACGTAAGC
TGTAAGTCTTGAC

>Ma01_t10610.1

ACCAGGGCACGTGCCTCACAATACCATGTATACTTAGATTTAAGAAAATGCTCCATGGCACATGCTGGCATACCATGTGTTTTTATGTCGGCACGTACTAAA
CCCGTA**CTGGTCCAACTGGTGACTGG**CATAGGTCCAGCACCAGAAACATCAAACCATGAACAAGGCAACAAAAGAAGTTTGATGTGTGTGTC

> Ma08_t27880.1

GTTTTGGGTTTGCTTGAACCCATGCCCTTCCAGTGCCAACAGAGGATCTGCTGTGTTGAAGCACCACTTTTGGGATGATGCATGTCTGGTCCGTGAATGTGGC
CAGATATTCTGTGGAGTTAGTCATCTG**ATGGAGACCATATGATCATA**ACCAAAAGACCTTTGGTTATTTAATCAGAAAGACATTATTCTCTCTTCCTAATGA
AGTTCTTTTAGTTTCTCCCAGATGGTAATTTCTTTGGCGACATGATGTTGCATTTTGATGGAAAATGTGGCCCCAGAAACGACATCCATGCTTGTGA

>ITC1587_Bchr5_T12273

AAAATTGGAGCCATCAGCACGTACCATAGAGCGGCATGCAGATTGACGGCAACCGAGGGTGGCATACCGATTGCTAAGAAGAGGCAGGCTATCCATG**GAG
CAAAGAAGAAGTTGA**CTCGGATGGGGTGATAGTACTCCCGGCGGATTGCTTCAAAGTAAAATATGATCTTCAGCACGTAGATGGCGGACACAATGCCCATC
AATGCAAGTGAGATGCACCAGAGAACGAGATTTACAGTCGGGCTTATATGCAGAAACGATGTGGATGGTGATGTAGCTAGTGTCTTCCACAGGATAGCTTG
GCT

>ITC1587_Bchr4_T08704
TGCTCATGACCCATTCATCATAACATAACAACAAAAACGGTGGTAGAAACAACAGTAATGAGAGGAAGGAAAAAGCAACAAGTGTGGCCCAACAATCTTC
CTATGAAGCTGATTAACGAGCTTGCTCGAGATCGGTAGTGCAGTGAAGGGCAGAAGTAGGCATCAACTACGACCGGAATTCTAGTAAGAGTTA**GCTGGAA**
CAACTGGTTCTGAATTCAGTGAGGCTGACTACTGACTGATCTAGTGGACTGGTCGATGCTTTTGATCGTGACAAAAGTCGTTAAGCAGCCTTAGTAGGAGAT
TGGGTGGAATCGAGCAGGCCTATA

>ITC1587_Bchr1_T00896
AGCAATCTCACGAAGGAGGAGTTTGTTCATGTTCTTCGACGCCAAAGCACCAGCTATCCTAGAGGAAGCTCCAAGTACAGGGGAGTGACTTTGCACAAATG
TGGAAAGTGGGAGGCCAGAATGGGCCAGTTACTGGGTAAAAAGTATATATACTTCTGGCCTTTCTTGCCAATAGATGAT**TGGAGTTCATATGTTTAT**AGAC
TAATGGTGTTGTGGATCATTCTGCTATGGTTTCTCTGGTTTAAAAGCATCGATTGTTCTCATAGGATTTCTTTGTCTGTGCTCTCAGATACGTCTATCTAGGTC
TTTTCGACACTGAAGTTGAAGCTGC
